# Hypothetical Rectal Microbicide Acceptability and Factors Influencing It among Men Who Have Sex with Men in Tianjin, China

**DOI:** 10.1371/journal.pone.0156561

**Published:** 2016-05-31

**Authors:** Guohong Zhang, Huifang Zhang, Hongxuyang Yu, Zheng Zhao, Jie Yang, Mianzhi Zhang, Minying Zhang

**Affiliations:** 1 School of Medicine, Nankai University, Tianjin, China; 2 Shenlan MSM Community Organization, Tianjin, China; 3 Tianjin Public Security Hospital, Tianjin, China; Shanghai Medical College, Fudan University, CHINA

## Abstract

**Objectives:**

To measure potential acceptability of rectal microbicides and to explore factors likely to affect their acceptability among men who have sex with men (MSM).

**Methods:**

Cross-sectional and retrospective surveys were conducted in this study. A questionnaire and a scale were used to measure the acceptability score for physical and functional characteristics of hypothetical rectal microbicides. We also evaluated the involvement of other factors such as sexual behaviors, social context, *etc*.

**Results:**

MSMs we interviewed showed a high acceptability to rectal microbicides, indicated by the mean acceptability score of 2.92 (SD, 0.54, scale of 1–4). The results also suggested that microbicides were preferred in a cream form that can moisten and lubricate the rectum, prevent HIV infection and go unnoticed by their partners. Multivariate analysis showed that the microbicides acceptability varied significantly by education level (*β* = 0.135; *P* = 0.028), having casual partners (*β* = 0.174; *P* = 0.007), frequency of lubricant use (*β* = 0.134; *P* = 0.031), history of HIV test (*β* = 0.129; *P* = 0.036), willingness to use lubricant (*β* = 0.126; *P* = 0.045), locus of control by partners regarding STI infection (*β* = 0.168; *P* = 0.009).

**Conclusions:**

A positive response to rectal microbicides among MSMs was found in our study, suggesting that rectal microbicides might have a potential market in MSMs and they might play an important role in HIV/STIs prevention as a supplement. Further studies may be considered to combine the acceptability study with clinical research together to understand the true feelings of MSMs when they use the products.

## Introduction

Recent epidemiological studies have confirmed that anal intercourse is widespread among MSMs both in the developed and developing world [1–3]. Unprotected anal intercourse is the common mode of HIV transmission among MSMs. Koblin *et al*. found that the men who reported unprotected receptive anal intercourse with partners were at a high risk of HIV infection, the attributable risk was 68.3%[4]. A previous study found the probability of HIV transmission by receptive anal intercourse was 18-fold higher than the probability of male to female transmission in penile-vaginal intercourse[5], which goes some way to explain why MSMs have been so disproportionately affected by HIV. Multiple sexual partners are popular among MSMs and they have various types of partners. With many MSMs marrying with women or keeping heterosexual partners, they have become the bridge for transmitting HIV to the female population, even the general population. MSM population has become the serious high-risk group of HIV infection. Condoms are technically effective to prevent the transmission of HIV, but the low level of condom use largely limits its preventive role in HIV transmission. To develop a protective alternative to condoms is urgently needed and of considerable practical and public health significance.

Microbicides are compounds that can be applied before sexual intercourse to eliminate or at least greatly reduce the risk of sexual transmission of HIV/STIs, and if effective products were found, which might have a great influence on HIV epidemic control[6–8].

To control HIV transmission, microbicides not only need to be efficacious against HIV, but also need to make the HIV high-risk population be willing to use them correctly and consistently[9–12]. The latter issue has been referred to as “acceptability”[11]. With the development of microbicides research, scientists have focused on microbicide acceptability, particularly on vaginal microbicides. The acceptability of vaginal microbicides has been explored globally over the past few years [13–20], but the acceptability of rectal microbicides has been insufficiently studied. Recently, with MSMs becoming the serious high-risk population of HIV infection, scientists have been paying more attention to the rectal microbicides, which are applicable for MSMs to prevent HIV spread [21]. With the promoting of vaginal microbicides study, rectal microbicides research has also made great progress. The existing studies have found that the acceptability of rectal microbicides was high among MSMs and lubricant formulations of rectal microbicides were more acceptable. Carballo-Dieguez found that, 94% of 307 MSM participants used lubricant when they had anal sex, 74% used lubricant at least in 80% of anal sex occasions, and 92% were willing to use lubricant if it could prevent HIV infection [6]. Kinsler *et al*. also found 77% of the participants reported the willingness of lubricant microbicides use to prevent HIV transmission [22]. Early studies on the acceptability of hypothetical rectal microbicides mainly focused on the preference to different characteristics of rectal microbicides, including color, smell, formulation and the influence on sexual intercourse *etc*.[23, 24]. Carballo-Dieguez *et al*. found the MSM population had different choices for hypothetical rectal microbicide formulations and use. Qualitative and clinical studies had also suggested that acceptable rectal microbicide formulations included gels, suppositories, and rectal douches [25, 26]. Besides, Carballo-Dieguez and his colleagues found that the acceptability of gels was higher than suppositories [9].

The acceptability of microbicides is not only influenced by their characteristics, but also influenced by demographic characteristics, high-risk behavior patterns and socio-cultural background. These factors might make the acceptability of microbicides diverse in different areas or populations and factors influencing the microbicide acceptability also varied by these factors. The high-risk behavior patterns and the particular socio-cultural background would influence the use of rectal microbicides among MSMs in China, so it is necessary to study the acceptability of hypothetical rectal microbicides, to understand how behaviors, environment and products’ characteristics to influence the acceptability before product promotion. Our study aimed to explore the factors likely to influence rectal microbicide acceptability among MSMs, and then to provide guidance for the development and promotion of related products.

## Methods

### Ethics issues

The study design and procedures were approved by the Institutional Review Board of Nankai University. Participants were fully informed about the aim and procedures of the study, and written informed consent was received from all participants. The interviews and the questionnaires were conducted anonymously, and all personal identifiers were removed from the final dataset to preserve participants’ privacy.

### Study Design and setting

This cross-sectional and retrospective study was conducted from March 2014 to December 2014 with quantitative method in Tianjin, China. Participant recruitment and interviews were conducted by the staff from Shenlan, the largest MSM community organization in Tianjin. A semi-structured questionnaire and an acceptability scale were used to assess the acceptability of microbicides and to explore the factors influencing the rectal microbicide acceptability among the participants through face to face interviews.

### Participants

MSMs went to Shenlan for HIV consultation and test were recruited in accordance with inclusion and exclusion criteria. Inclusion criteria included: (1) male with self-identification as gay or bisexual, (2) current residence in Tianjin, (3) had sexual intercourse with men within the last six months, (4) could understand and communicate in mandarin or the local dialect. Exclusion criteria included: (1) had hearing or language barrier, (2) suffered from mental illness, (3) and couldn’t cooperate with our study.

### Interviews and measures

Before interview, research objectives and related content were fully explained to the eligible MSMs. Then the MSMs signed informed consent if they agreed to participate in the study and proceeded to complete the survey. The survey was carried out in a separate room by face-to-face interview using a semi-structured questionnaire and an acceptability scale. Further explanation was given if the participants had any question at any time during the interview, and each interview lasted approximately 20 to 30 minutes.

The semi-structured questionnaire was designed to collect information on demographic characteristics (age, ethnicity, native place, marital status, education level, professional status, cohabitation status, and average monthly income), sex behavior characteristics (site or way of homosexual partner seeking, number of sex partners, partner types, frequency of sexual intercourse, lubricants and condom use in recent month, *etc*.), attitudes towards HIV/STIs prevention, perceived risk and the relevant factors (experience of HIV test, substance use and STIs infections, *etc*.) associated with HIV/STIs among MSMs. The questionnaires were completed by face-to-face interview anonymously.

Attitudes towards HIV/STIs prevention and perceived HIV/STIs risk were assessed by 3 questions (whether you worried about sexually transmitted infection, whether you worried about HIV infection, the possibility of infecting HIV) and a scale which included 8 items that involved controls to prevent HIV/STIs. The scale included 3 dimensions: (1) an internal locus of control (3 items) that assessed the level to which the participants believed that HIV/STIs infection was determined by their own behavior; (2) a partner locus of control (2 items) that assessed the level to which the participants believed that HIV/STIs infection was determined by their partners; and (3) a chance locus of control (3 items) that assessed the level to which the participants believed that HIV/STIs was determined by fate. Each item was rated on a 4-point Likert scale (1 = strongly disagree, 2 = disagree, 3 = agree, 4 = strongly agree).

The hypothetical rectal microbicide acceptability scale we used was adapted from Weeks’ study [17] on the acceptability of vaginal microbicides among women and the scale included 20 items mainly about the microbicides’ characteristics and functional features. The characteristics and functional features included formulations, ways to insert, smell, time of action initiation, working time, price, timing of insertion, lubricity, effectiveness, side effects, *etc*. The participants completed each item privately in the examination room and their reactions to hypothetical products were rated on a 4-point Likert scale (1 = completely unacceptable, 2 = somewhat unacceptable, 3 = somewhat acceptable, 4 = completely acceptable). The Cronbach’s alpha for this scale was 0.90, demonstrating a high internal consistency. Means of all scores of the 20 items were obtained to assess the levels of acceptability, and higher mean score indicated higher levels of rectal microbicide acceptability.

### Data Analysis

Descriptive statistics were used to summarize demographic characteristics, sexual behavior characteristics, attitude toward HIV/STIs, perceived HIV/STI risk, and the scores of microbicide acceptability. Microbicide acceptability was evaluated by the means of all scores of the 20 items in the scale. One-way ANOVA and t-test were conducted as univariate analysis to identify continuous variables influencing microbicide acceptability. Categorical variables were presented as number and were compared using chi-square test. Based on the univariate analysis results, multiple linear regression analysis was constructed with statistically significant independent variables to calculate the standardized regression coefficient (*β*) for the exploration of the factors associated with the acceptability of rectal microbicides, with rectal microbicide acceptability scores being dependent variable and other factors being independent variables. Confidence intervals (CIs) were presented as 95% CIs and *p*-values less than 0.05 were deemed statistically significant.

Statistical analysis was performed using Stata software (Version 12, College Station, TX, USA).

## Results

A total of 356 participants completed the questionnaires. Among them, 6 unqualified questionnaires were excluded, including 4 uncompleted questionnaires and 2 questionnaires with randomly or incoherent responses. A total of 350 qualified questionnaires were obtained (S1 Compressed File).

### Demographic and sexual behavior characteristics

The mean age of the participants was 27.66 ± 7.76 years with a range from 16 to 54, and most of them (75.4%) were unmarried. The overall education level was high, with 62.3% of the participants receiving college education or higher. The vast majority of the participants (70.9%) reported they hadn’t a cohabiting partner. The average monthly income for 41.1% of the participants was between 2000 and 4000 China Yuan (CNY), most of those reported no earning (79.3%) were students. Demographic and sexual behavior characteristics of the participants were presented in Table 1.

**Table 1:** Demographic characteristics and sexual behavior.

Characteristics	Participants *N (%)*
**Demographic characteristics**	
**Age(years)**	
16~	183(52.3)
26~	112(32.0)
36~54	55(15.7)
**Education level**	
Middle school or lower	34(9.7)
High school	98(28.0)
College or higher	218(62.3)
**Marital status**	
Single	264(75.4)
Married	67(19.1)
Divorced/separated/widowed	19(5.4)
**Average monthly income**(CNY)	
0	82(23.4)
<2000	34(9.7)
2000~	144(41.1)
4000~	90(25.7)
**Cohabitation status**	
No cohabiting partner	248(70.9)
Cohabiting with male	57(16.3)
Cohabiting with female	39(11.1)
Cohabiting with both male and female	6(1.7)
**Sexual behavior**	
**Site or way of homosexual partner seeking**	
Bar	11(3.1)
Commercial bath center	41(11.7)
Park	16(4.6)
Internet	244(69.7)
Others	38(10.9)
**Having sex with casual male in recent month**	
Yes	151(43.1)
No	199(56.9)
**Participating in group sex**	
Yes	51(14.6)
No	299(85.4)
**Frequency of lubricant use in the past month**	
Never	12(4.8)
Occasionally	24(9.6)
Most of the time	29(11.6)
Every time	185(74.0)
**Total**	350(100.0)

We classified sites or ways of homosexual partner seeking in this study as bar, commercial bath center, park, Internet and others. Sex partners were classified as primary partner, casual partner, and commercial partner. The majority of the participants (69.7%) reported looking for partner on the Internet, and 56.9% reported having sex at least once with casual male partner during the preceding month. About 14.6% of the participants reported participation in group sex and 74.0% reported using lubricant consistently during intercourse in the preceding month. Only 4.8% of the participants had never used lubricant during the preceding month.

### Awareness, attitudes and behaviors towards HIV/STIs prevention and willingness of lubricant use

Among 350 participants, 27 (7.7%) had been infected with HIV, and 80.9% were worried about being infected with HIV, whereas nearly half of the participants (47.1%) believed that the chance of being infected was little. The participants who believed their behaviors were in the control of their risks for HIV/STIs infection scored the highest on the acceptability scale (2.77; SD, 0.45). Vast majority of the participants (95.7%) were aware that condom could prevent HIV transmission effectively, meanwhile 87.7% reported that they were willing to use condoms. Lubricant use preference was reported by 93.1% of the participants. Among all participants, 75.1% reported ever being tested for HIV. The specific results were shown in Table 2.

**Table 2:** Awareness, attitudes and behaviors towards HIV/STIs preventing and willingness of lubricant use.

Characteristics	*N (%) /*$\bar{\text{x}}$±s
**Perceived possibility of infecting HIV**	
Strong possibility	38(10.9)
Little possibility	165(47.1)
Impossibility	33(9.4)
Have no idea	87(24.9)
Had been Infected	27(7.7)
**Awareness of preventing HIV/STIs** ($\bar{x}$**±s)**	
Internal locus of control	2.77±0.50
Locus of control by partners	2.68±0.57
Locus of control by chance	1.82±0.58
**Willingness to use lubricant**	
Yes	326(93.1)
No	24(6.9)
**Condom use to prevent HIV/STIs in recent 6 months**	
Yes	238(68.0)
No	112(32.0)
**Experience of HIV test**	
Yes	263(75.1)
No	87(24.9)
**Total**	350(100.0)

### Rectal microbicide scale scores

Results of our study showed a high acceptability to rectal microbicides among MSMs, indicated by the average scale score of 2.92 (SD, 0.54, scale of 1–4). The mean score of microbicide acceptability scale was listed in Table 3. The scale had high internal consistency, Cronbach’s *α* = 0.926.

**Table 3:** Rectal microbicide acceptability scale scores.

Items	Scores($\bar{\text{x}}$±s)
If the microbicide is formulated as a tablet or suppository	3.10±0.80
If the microbicide leaks out after intercourse	2.85±0.85
If the microbicide makes your rectum wetter than normal during sex	3.33±0.68
If the microbicide is formulated as cream	3.18±0.80
If the microbicide is formulated liking jelly	2.87±0.86
If the microbicide needs to be inserted into the rectum 15 minutes before sex	2.96±0.83
If the microbicide makes your rectum drier during sex	2.21±0.84
If the microbicide needs to be put in rectum with your finger	2.79±0.88
If the microbicide needs to be inserted into the rectum every time before sex	2.91±0.78
If the microbicide needs to be injected into rectum	2.44±0.96
If the microbicide has no noticeable smell	3.19±0.81
If the microbicide causes slight uncomfortable	2.39±0.84
If the microbicide is not noticeable to your sexual partner	3.19±0.79
In order to make the microbicide have better effect, you can’t douched until several hours later after sex	2.62±0.89
If the microbicide has a pleasant smell	3.11±0.88
If one application of the microbicide lasts multiple times of sexual intercourse in one day	3.13±0.13
If the microbicide leaks out after insertion before sex	2.79±0.84
If the microbicide can prevent HIV infection	3.46±0.67
If the microbicide is formulated as foam	2.88±0.87
If the microbicide is formulated as capsule	3.02±0.83
**Mean score of all items**	**2.92±0.54**

For the formulations of rectal microbicides, the acceptability score of cream formulation (3.18±0.80) was higher than that of tablets or suppositories (3.10±0.80), and jelly formulation (2.87±0.86) acceptability score was the lowest. Both the microbicides without a noticeable smell (3.19±0.79) and those with a pleasant smell (3.11±0.88) had high acceptability scores.

For the application of rectal microbicides, the participants prefer putting rectal microbicides with a finger (the acceptability score was 2.79±0.88) to injecting into rectum (2.44±0.96). The microbicide acceptability score of inserting into the rectum 15 minutes before sex intercourse was 2.96 (SD, 0.83).

For the function of rectal microbicides, the acceptability score of the microbicides being efficacious for multiple sexual intercourses in one day was high (3.13±0.13). If the microbicides could make the rectum wetter than normal during sexual intercourse, the participants preferred to accept them (3.33±0.68), compared with those making rectum drier which had the lowest mean score (2.21±0.84). Most participants would like to use the microbicides if they were not noticeable to their sexual partners (3.19±0.79). The mean score of microbicide acceptability that could prevent HIV infection was 3.46(SD, 0.67), with 54.6% of the participants reported being completely acceptable, and 38.0% reported being somewhat acceptable.

### Univariate analysis of factors related to the rectal microbicide acceptability

Univariate analysis showed that the mean score of rectal microbicide acceptability varied significantly by education level (*F* = 9.20, *P*<0.000) and income level *(F* = 2.71, *P* = 0.045). No statistically significant difference was found among different age (χ^2^ = 5.12, P = 0.077) or marital status (*F* = 0.39, *P* = 0.678) of the participants. *Post-hoc* analyses results indicated that the participants who received college education or higher had higher scores than those who only received high school education (*P* = 0.008) and middle school education or even lower (*P*<0.000). The participants whose average monthly income were 4000 CNY or more had higher scores than those with monthly income of 2000 to 4000 CNY (*P* = 0.043) and less than 2000 CNY (*P* = 0.021), but there was no statistically significant difference was found between those with monthly income of 4000 CNY or higher and those with no income, and the rectal microbicide acceptability scores of those with no income was higher than those with monthly income of less than 2000 CNY (*P* = 0.046).

According to the univariate analysis, the microbicide acceptability scores also varied significantly by experience of having sex with casual partners (*t* = -2.57, *P* = 0.011), participating in group sex (*t* = -2.34, *P* = 0.020), the sites or ways of homosexual partner seeking (*F* = 3.51, *P* = 0.008) and frequency of lubricant use (*F* = 3.28, *P* = 0.022). The mean score of acceptability among the participants who had sex with casual partners in recent month or participated in group sex was higher than those didn’t. *Post-hoc* analyses showed that the participants who sought homosexual partner in commercial bath center (*P* = 0.043) or on the Internet (*P* = 0.002) were more willing to accept the rectal microbicides than those who sought partner in bar, but there was no significant difference among other groups. The mean score of rectal microbicide acceptability among the participants who used lubricant during every sexual intercourse was higher than that of who never used lubricant (*P* = 0.019) or used lubricant occasionally (*P* = 0.027).

Microbicide acceptability scores were found also varied by the perceived possibility of being infected with HIV (*F* = 3.08, *P* = 0.016), willingness of condom use to prevent HIV/STIs (*t* = -2.43, *P* = 0.015), willingness of lubricant use (*t* = -3.55, *P*<0.000) and experience of HIV antibody test (*t* = -3.03, *P* = 0.003). The acceptability scores among the participants who had used condoms to prevent HIV/STIs were higher than those who hadn’t. The mean score of acceptability among the participants who had been tested for HIV was higher than those who had not. The participants who were willing to use a lubricant during sexual intercourse were more willing to accept rectal microbicides than those who were not. *Post-hoc* analyses revealed that the participants who perceived strong possibility of HIV infection had higher scores than those who thought it was impossible to be infected (*P* = 0.026). And the participants who thought there was little possibility of HIV infection had higher scores than those who thought it was impossible to be infected (*P* = 0.013) and those who had no idea about whether they could be infected with HIV (*P* = 0.018). As for the HIV/STIs locus of control, a positive correlation was found between the mean score of the microbicide acceptability and the HIV/STIs infection locus of control by partners(*R* = 0.17, *P* = 0.001). Results of univariate analyses were shown in Table 4.

**Table 4:** Univariate analyses of mean scores for rectal microbicide acceptability.

Variables	*N (%) /*$\bar{\text{x}}$±s	Score($\bar{\text{x}}$±s)	*F/t/R*	*P*
**Demographic characteristics**				
**Educational level**				
Middle school or lower	34(9.7)	2.63±0.49	9.20^b^	0.000
High school	98(28.0)	2.83±0.51		
College or higher	218(62.3)	3.00±0.53		
**Average monthly income (CNY)**				
0	82(23.4)	2.98±0.58	2.71^b^	0.045
<2000	34(9.7)	2.76±0.56		
2000~	144(41.1)	2.87±0.55		
4000~	90(25.7)	3.01±0.43		
**Sex behavior**				
**Site or way of homosexual partner seeking**				
Bar	11(3.1)	2.47±0.58	3.51^b^	0.008
Commercial bath center	41(11.7)	2.84±0.46		
Park	16(4.6)	2.80±0.57		
Internet	244(69.7)	2.98±0.53		
Others	38(10.9)	2.82±0.54		
**Having sex with casual partner in recent month**				
Yes	151(43.1)	3.00±0.54	-2.57^a^	0.011
No	199(56.9)	2.86±0.52		
**Participating in group sex**				
Yes	51(14.6)	3.08±0.59	-2.34^a^	0.020
No	299(85.4)	2.89±0.52		
**Frequency of lubricant use in past month**				
Never	12(4.8)	2.60±0.45	3.28^b^	0.022
Occasionally	24(9.6)	2.71±0.48		
Most of the time	29(11.6)	2.89±0.47		
Every time	185(74.0)	2.97±0.55		
**Perceived possibility of HIV infection**				
Strong possibility	38(10.9)	3.03±0.58	3.08^b^	0.016
Little possibility	165(47.1)	3.00±0.51		
Impossibility	33(9.4)	2.74±0.52		
Have no idea	87(24.9)	2.83±0.55		
Have been Infected	27(7.7)	2.81±0.52		
**Attitudes towards preventing HIV/STIs and willingness of lubricant use**				
**Willing to use lubricant**				
Yes	326(93.1)	2.95±0.53	-3.55^a^	0.000
No	24(6.9)	2.55±0.49		
**Use condom to prevent HIV/STIs in recent 6 months**				
Yes	238(68.0)	2.97±0.52	-2.43^a^	0.015
No	112(32.0)	2.82±0.55		
**Experience of HIV test**				
Yes	263(75.1)	2.97±0.50	-2.70^a^	0.008
No	87(24.9)	2.77±0.61		
**Awareness of HIV/STIs prevention(** $\bar{x}$**±s)**				
Internal locus of control	2.77±0.50	2.92±0.54	0.09^c^	0.090
Locus of control by partners	2.68±0.57	2.92±0.54	0.17^c^	0.001
Locus of control by chance	1.82±0.58	2.92±0.54	-0.03^c^	0.508

^a^*t*-test

^b^one-way ANOVA

^c^Spearman rank correlation analysis

### The willingness of rectal microbicide use when having sex with different type of partners

Results of our study showed the participants preferred to use rectal microbicides with casual partners rather than primary partners or commercial partners. The results also revealed that 70.8% of the participants were willing to use microbicides with casual partners, meanwhile primary partners and commercial partners were 63.9% and 59.0%, respectively. Compared by chi-square test, results demonstrated when the participants had sex with different type partners, the willingness of rectal microbicide use was statistically significant (*χ*^*2*^ = 13.74, *P* = 0.008). Statistical significant difference of rectal microbicide use willingness was found by post-hoc analyses between primary partners and commercial partners (*χ*^*2*^ = 8.56, *P* = 0.014), casual partners and commercial partners (*χ*^*2*^ = 8.47, *P* = 0.014). But no significant difference was found between primary partners and casual partners (*χ*^*2*^ = 3.35, *P* = 0.187).The specific results were shown in Table 5.

**Table 5:** The willingness of rectal microbicide use with different type of partners.

use intention	Commercial partners	Primary partner	Casual partners	*χ*^*2*^	*P*
Be willing to	49(59.0)	195(63.9)	179(70.8)	13.74	0.008
Maybe willing to	21(25.3)	92(30.2)	59(23.3)		
Be not willing to	13(15.7)	18(5.9)	15(5.9)		

### Multivariate analysis of factors influencing the rectal microbicide acceptability

Based on the univariate analysis results, multivariate linear regression analysis model were constructed with statistically significant independent variables to analyze those factors influencing the rectal microbicide acceptability. The results were shown in Table 6.

**Table 6:** Linear regression of factors influencing rectal microbicide acceptability.

variables	*B*	*SE*	*β*	*P*
Constant	0.806	0.314		0.011
Education level	0.097	0.044	0.135	0.028
Having casual partners in recent month	0.188	0.069	0.174	0.007
Frequency of lubricant use in past month	0.084	0.039	0.134	0.031
Locus of control by partners	0.157	0.056	0.169	0.006
Experience of HIV test	0.158	0.075	0.129	0.036
Willing to use lubricant	0.250	0.124	0.126	0.045

*R*^*2*^ = 0.251, *P*<0.001.

Multivariate analysis showed that the rectal microbicide acceptability scores varied significantly by education level, having casual partners, experience of lubricant use, experience of HIV test, willingness of lubricant use and locus of control by partners. The scores of microbicide acceptability increased with the level of education (*β* = 0.135, *P* = 0.028). The participants who had casual male partners during the preceding month had higher scores than those who hadn’t (*β* = 0.174, *P* = 0.007). The acceptability scores increased with the frequency of lubricant use during the preceding month (*β* = 0.134, *P* = 0.031).The microbicide acceptability scores among the participants who had been tested for HIV was higher than among those who had not (*β* = 0.129, *P* = 0.036). The scores of microbicide acceptability showed a positive correlation with the mean scores of HIV/STIs locus of control by partners (*β* = 0.169, *P* = 0.006). And the participants who were willing to use a lubricant during sexual intercourse were more willing to accept the rectal microbicides (*β* = 0.126, *P* = 0.045).

### Demand for rectal microbicides

Results of our study showed if the microbicides were efficacious to prevent HIV/STIs, nearly half of the participants (48.6%) would be willing to buy them. For the price of rectal microbicides, 39.4% of the participants chose 10~20 CNY per sex intercourse, and 24.0% of the participants chose less than 3 CNY per sex intercourse. For the working time of rectal microbicides, about 36.3% of the participants chose more than 10 hours, and 27.4% of the participants chose 2~5 hours. More than half of the participants (55.1%) hoped that microbicides were over the counter (OTC).

We also found that 44.0% of the participants were concerned about the effectiveness of rectal microbicides to prevent HIV/STIs, about the side effects and the safety of rectal microbicides were 24.0% and 23.7% of the participants, respectively.

## Discussion

Research from the University of London indicated that about 2.5 million recently HIV-infected cases globally could be prevented in just three years even if the microbicides are effective at a level of 60% in HIV transmission [27]. Microbicides can effectively play their role in HIV prevention only if the high-risk population was willing to use them correctly and consistently, so it is necessary to study the acceptability of microbicides among target population.

This study aims to measure the potential acceptability of rectal microbicides and the factors likely to influence it among MSMs. Due to the sensitivity and specificity of MSMs, participant recruitment and interview were conducted by the staff of Shenlan as the role of peers which could largely improve the authenticity of information[28]. MSMs we interviewed showed a high acceptability to rectal microbicides, and positive acceptability to microbicides had been revealed in HIV/AIDS high-risk populations in many studies [13, 18, 29, 30].

Regarding preference for the physical characteristics of microbicides, the participants in our study highly accepted the microbicides in a cream form that could moisten and lubricate the rectum, therefore might enhance sexual pleasure and relieve the agonies of sexual intercourse. Sexual pleasure has been proposed as an important consideration associated with microbicide acceptability [31, 32]. In Montgomery’s and Stadler’s studies among women and their partners, majority of participants would accept the microbicides which could increase sexual pleasure and make vagina wetter [33, 34]. Furthermore, Carballo-Diéguez et al. found that 93% of the MSMs used lubricants regardless of condom use among those having anal sex in the past year, and 92% were willing to use a lubricant with an anti-HIV microbicidal agent [6]. Results of Kinsler’s study on lubricant use among MSMs in Peru also showed that 77% of the participants would like to use a lubricant to prevent HIV transmission [22].Being familiar to the participants, microbicides in a cream form that can moisten and lubricate the rectum were easily accepted.

It was also revealed that most participants in our study preferred the microbicides which were not noticeable to their sexual partners, indicating that they would like to use it covertly, similar to the study on microbicide acceptability among FSWs in Beijing, China [18]. The participants in our study showed a high acceptability to the microbicides without smell or with a pleasant smell, which was different from Han’s study in which the female sex worker participants had showed a preference for the microbicides without smell [18]. The difference was perhaps due to the wide and frequent use of lubricant with pleasant smell among MSMs. So, even if the microbicides had a pleasant smell, sexual partners were also not easily to be aware of the use. However, lubricants, especially those with a smell, were not so prevalent among FSWs, so FSWs showed a high acceptability to the microbicides without smell [18]. Although the above results showed a positive response to microbicides among MSMs and FSWs in China, they would definitely prefer not to tell their partners their use of microbicides. However, a number of studies from foreign countries reported that participants, including MSMs and FSWs, would prefer to tell their partners the use of microbicides to preclude their partners from thinking they were unfaithful [13, 35–37]. The difference suggested that Chinese special social and cultural background and partner relationship would affect the microbicide use. In China, men played a dominant role in family and heterosexual relationship, women found it difficult to negotiate with their partners as to appropriate protective action they might wish to take, therefore they preferred to use the microbicides covertly [18, 38]. As for homosexual relationship, a hidden epidemic of intimate partner violence (IPV) had been revealed among MSMs in China. Davis *et al*. found, among 610 participants, 29.8% reported experiencing at least 1 type of IPV [39]. In Dunkle’s study, 51% of the MSMs reported emotional, physical, or sexual abuse from a male sexual partner. Money boys reported even more overall abuse than did other MSMs [40]. IPV found among MSMs indicates inequality in homosexual relationships, therefore MSMs who felt weak in the relationship might also prefer to use microbicides covertly to avoid violence.

Our study also showed that most MSMs had a high acceptability to the microbicides which could prevent HIV infection, indicating the effect of microbicides to prevent HIV would directly affect the use of microbicides among MSMs. The study of Kinsler *et al*. also found the preference for the rectal microbicides with 80% effectiveness vs. 40% effectiveness [41]. So it was important to improve the effect of HIV prevention during future development and promotion of microbicides. It was reported that chemically modified proteins exhibited potent anti-HIV activities and antiviral activity against infection by human papilloma viruses (HPV), which has great potential for further development as a microbicide to prevent the anal HIV infection and HIV/HPV coinfection [42–45]. Considering the high coinfection of HIV/HPV among MSMs, if so effective microbicides were available, the target population should highly accept them. However, further research about the actual interest in the chemically modified proteins as microbicides is needed.

A positive correlation between microbicide acceptability and education level was found in our study, which conflicted with Han’s study among FSWs [38]. The conflict might be caused by condom use. Condom use increased with the level of education among FSWs [46], and had a negative influence on the microbicide acceptability among FSWs. However, in our study, condom use had no significant influence on the microbicide acceptability among MSMs. The study of Weeks *et al*. also indicated education level had significant influence on the microbicide acceptability [17]. Hence the influence of education level on the microbicide acceptability is still ambiguous and further research is expected.

A significant positive correlation was also found between microbicide acceptability and the experience of having sex with casual partners in the past month. Perhaps those MSMs perceived a relative higher risk. Giguere *et al*. also found that MSMs expressed high likelihood of microbicide use with one-night stands, whom they perceived as riskier [47]. This indicating that rectal microbicides might have a potential market among MSMs who had sex with casual partners and it might play an important role in HIV/STIs prevention as a supplement.

We also found the participants who were willing to use lubricants during sexual intercourse were more willing to accept the rectal microbicides than those who were not, and the microbicide acceptability had a significant positive correlation with the frequency of lubricant use. Kinsler’s study got the similar result (OR = 1.96) [22]. The results indicated that the participants who were frequently used lubricants would prefer to accept the microbicides, and we should give much advance publicity to microbicides among the MSMs who were not willing to use lubricants in the future.

In our study, the participants had been tested for HIV had higher acceptability than those who had not. Perhaps, because the participants, who had been tested for HIV, perceived the risk of HIV infection, learned more knowledge about HIV and paid more attention to their health and the prevention of HIV/STIs. So they were more likely to accept the rectal microbicides. Previous studies also revealed that perceived risk of HIV was associated with microbicide acceptability [30, 48]. The results of our study indicated perceived risk of HIV was conducive to change the HIV-related attitudes and increase the understanding of HIV, so as to encourage them to take measures to prevent HIV, and much advance publicity and education on HIV should be given to MSMs who had low perceived risk of HIV during future development and promotion of microbicides.

A significant positive correlation between locus of control by partners and microbicide acceptability was revealed, which was similar with Wang’s study in China [30]. This indicated that the MSMs who tend to believe their partners were in control of their HIV/STIs risks were more likely to accept microbicides, which suggested that locus of control by partners might be an important indicator of microbicide use. We need to fully understand the attitude of MSMs toward HIV/STIs infection to improve microbicide use in the future promotion of the products.

So far, most studies on acceptability of rectal microbicides were conducted in the United States and focused on products characteristics [9, 23, 25, 26]. Our study not only measured the product characteristics and function, but also participants’ social and behavioral factors. Many factors were found being associate with microbicide acceptability, including education level, the experience of having sex with casual partners in the past month, frequency and willingness of lubricant use, experience of HIV test, locus of control by partners *etc*. But the variation in these variables only accounted for 25.1% of the overall variation in microbicide acceptability, indicating some of the factors influencing microbicide acceptability were still undiscovered. Our research proposed that social and behavioral factors should be taken into full account in microbicide promotion. Behavioral and social science research was proposed to support both clinical trial performance and promotion of future microbicide products [49, 50]. Further studies might be considered to combine the acceptability study with clinical research together to understand the true feelings of MSMs when they use the products, and then to provide guidance for the development and promotion of rectal microbicides with high acceptability and adherence.

Several limitations must be considered in our study. First, information was collected by the participants’ self-reporting in our study. Given that the questionnaire involved some items on sensitive information of their sexual behaviors, the data obtained in our study might be under reported. Also, the rectal microbicides are still underdevelopment and no efficacious products are available. The “acceptability” in our study for hypothetical microbicides might not reflect actual interest in microbicide products entirely. But our study could provide the basis for the further development and promotion of related products.

## Conclusion

Results of our study showed a high acceptability to rectal microbicides among MSMs, suggesting that rectal microbicides might have a potential market in MSMs and play an important role in HIV/STIs prevention as a supplement. The microbicide acceptability varied significantly by education level, having sex with casual male, frequency of lubricant use, experience of HIV test, willingness of lubricant use, locus of control by partners and characteristics of the products, which should be taken into account during future development and promotion of microbicides.

## Supporting Information

S1 Compressed File Data set of the study. (RAR)
